# Recognition of Electroencephalography-Related Features of Neuronal Network Organization in Patients With Schizophrenia Using the Generalized Choquet Integrals

**DOI:** 10.3389/fninf.2021.744355

**Published:** 2021-12-14

**Authors:** Małgorzata Plechawska-Wójcik, Paweł Karczmarek, Paweł Krukow, Monika Kaczorowska, Mikhail Tokovarov, Kamil Jonak

**Affiliations:** ^1^Department of Computer Science, Lublin University of Technology, Lublin, Poland; ^2^Department of Clinical Neuropsychiatry, Medical University of Lublin, Lublin, Poland

**Keywords:** schizophrenia, extended Choquet integral, classifiers, aggregation, Sugeno fuzzy measure

## Abstract

In this study, we focused on the verification of suitable aggregation operators enabling accurate differentiation of selected neurophysiological features extracted from resting-state electroencephalographic recordings of patients who were diagnosed with schizophrenia (SZ) or healthy controls (HC). We built the Choquet integral-based operators using traditional classification results as an input to the procedure of establishing the fuzzy measure densities. The dataset applied in the study was a collection of variables characterizing the organization of the neural networks computed using the minimum spanning tree (MST) algorithms obtained from signal-spaced functional connectivity indicators and calculated separately for predefined frequency bands using classical linear Granger causality (GC) measure. In the series of numerical experiments, we reported the results of classification obtained using numerous generalizations of the Choquet integral and other aggregation functions, which were tested to find the most appropriate ones. The obtained results demonstrate that the classification accuracy can be increased by 1.81% using the extended versions of the Choquet integral called in the literature, namely, generalized Choquet integral or pre-aggregation operators.

## Introduction

Mental illnesses are usually long-lasting conditions associated with great psychological suffering, the substantially limited possibility of independent functioning, and social development. Among them, schizophrenia (SZ) is one of the most severe forms of mental health disorder with the complex and multidimensional clinical picture. The onset of SZ occurs most often in adolescence or early adulthood commonly has a slow and hidden course consisting of gradual augmenting of the so-called negative syndromes, i.e., loss of interests, affective blunting, reduced initiative, and social isolation, and more or less delayed phase of active psychotic exacerbation characterized by the presence of delusions, i.e., incorrect judgments of reality and the behavior of other people, as well as hallucinations, i.e., incorrect sensory impressions, most often in the auditory form (Rosen et al., 1984; Heilbronner et al., 2016). In addition to the negative and positive symptoms, there are also various cognitive disorders including disturbances in the course of thinking and deficits in specific cognitive domains, such as attention, memory, cognitive speed, language, and communication, and difficulties with adapting to new circumstances and problem-solving (Szöke et al., 2008; Krukow et al., 2017; Green et al., 2019). It should be noted that long-term pharmacological treatment of the disease is the main form of therapeutic intervention focused mainly on psychotic syndromes. However, even when modern methods of treatment are applied, distortions of cognitive processes improve to a much lesser extent, often causing lifelong constraints in achieving full independence (Keefe, 2019). Personal, social, and economic burdens associated with severe mental illness prompt researchers to search for new therapies and also to develop accurate methods of differential diagnosis, which should be ultimately based on objective, biological markers (Pantelis et al., 2009). The development of new neuroimaging techniques enables researchers to identify neural circuits that underline the human brain integration system. Various neuropsychiatric conditions are correlated with changes in brain communication patterns and pointed as potentially useful biomarkers for clinical applications (Sporns et al., 2005). In accordance with the results of earlier studies focused on brain synchronization, SZ is seen unequivocally as a disconnectivity disorder characterized by abnormal functional and structural connectivity of the brain (Friston and Frith, 1995). Application of diffusion tensor imaging (DTI) methods, such as magnetic resonance imaging (MRI) technique, into SZ research, showed disconnection and multiple microstructural aberrances of brain white matter fibers (Zalesky et al., 2011; Klauser et al., 2017). Studies based on electroencephalography (EEG) and functional MRI (fMRI) also revealed abnormalities in the functional connectivity of the brain, which were also correlated with the clinical picture of the SZ (Skudlarski et al., 2010; Uhlhaas, 2013; Krukow et al., 2018). Nevertheless, to understand the systemic level of the brain organization and to explain neurophysiological processes such as disconnectivity syndrome in the SZ, researchers started to analyze the brain as a complex network (van den Heuvel and Sporns, 2013). The neural network is understood as a system of spatial (anatomical) and temporal (synchronous firing of neuronal assemblies) dimensions, involving different brain regions interconnected with each other (Zalesky et al., 2010). However, to analyze the state of the functional and structural connections from the viewpoint of the entire brain, an infinite number of potential anatomical and functional interactions between a given set of neural regions makes such an analysis a challenge almost impossible to obtain. Therefore, a graph theorem has been introduced to solve this problem and to test the complex whole-brain networks in their global dimension (Van Den Heuvel and Fornito, 2014). Previous studies investigating the neural brain networks in SZ showed significantly changed network organization as indicated by graph-analytical measures of global, short communication paths (Yan et al., 2015), local organization (Alexander-Bloch et al., 2010), and small-worldness (balance between local segregation and global integration) (Shim et al., 2014). Aberrant functional networks in the SZ were also linked with cognitive impairments (Sheffield et al., 2015; Krukow et al., 2020) and the duration of the illness (Jonak et al., 2019).

Previous studies considered the problem of automated classification of altered brain activity in SZ based on the EEG or fMRI data. Among traditional classifiers, methods such as support vector machine (SVM; Shim et al., 2016; Liu et al., 2017; Huang et al., 2018), adaptive boosting (Sabeti et al., 2011), kernel discriminant analysis (KDA; Zhu et al., 2018), or nearest neighbor algorithm (Parvinnia et al., 2014) are used. Some of these studies (Sabeti et al., 2011; Parvinnia et al., 2014) applied time-frequency features obtained from single EEG channels, which is a limited capacity approach as it does not consider interactions between channels understood as a network. Other authors applied a convolutional neural network (Phang et al., 2019) and deep neural networks (DNNs; Plis et al., 2014; Guo et al., 2017). In addition, manifold learning for aggregation was considered in works by Shen et al. (2010); Anderson and Cohen (2013), and Gallos et al. (2021a, b). The idea of applying fuzzy classification into SZ-based data is a relatively new concept, as there are only a few papers on this subject (Sabeti et al., 2007; Silvana et al., 2018). One of the answers to the problems related to the application of single classifiers in the processes of automated disease diagnosis may be using various aggregation models. Aggregation can be carried out at the stage of data analysis in the form of information fusion and the stage of analysis of classification results. Despite some shortcomings such as extending the duration of the diagnosis process or the need to implement additional algorithms, the undoubted advantage of this approach is the increase in the effectiveness of classification, which, combined with the field of application critical to human health, is of key importance. Common examples of aggregation operators are voting, maximum, minimum, and median functions. The methods based on triangular norms (Klement et al., 2000) or ordered weighted averaging operators (OWA; Yager and Kacprzyk, 2012) are somewhat more complex. Various general approaches to the aggregation of classifiers were already presented (e.g., in publications of Alsina et al., 2006; Beliakov et al., 2007; Grabisch et al., 2009; Calvo et al., 2012; Gągolewski, 2015; Dolecki et al., 2016; Baczyński et al., 2017). Recently, one of the dominant techniques is using the Choquet integral or its generalizations or extensions (Kwak and Pedrycz, 2004, 2005; Karczmarek et al., 2014, 2017a, b, 2018, 2019b; Anderson et al., 2018; Rutkowska et al., 2020). In particular, recent studies on the so-called pre-aggregation functions offer hope for the development of this approach (Lucca et al., 2015, 2016, 2017; Bustince et al., 2016; Dimuro et al., 2017; Dias et al., 2018). They are particularly used in computer image analysis and its subdiscipline of facial recognition (Karczmarek, 2018; Karczmarek et al., 2019a). Detailed theoretical and practical analyses of the approach based on pre-aggregation functions, i.e., slightly weakening the classical aggregation (Beliakov et al., 2007) conditions, are still ongoing. Nevertheless, the weakening of these conditions does not have a negative impact on the classification results, which is confirmed by the experimental outcomes from the above-mentioned studies. A survey of the generalizations of Choquet integral can be found in Dimuro et al. (2020).

The problem undertaken in this study was related to the effective automatic distinction between patients diagnosed with SZ and healthy subjects based on EEG-based features of neuronal network organization. The main goal of this study was to find the appropriate operator aggregating the neurophysiological outcomes and categorizing them as patients diagnosed with SZ or healthy controls (HC), i.e., increasing the effectiveness of the classification. For this purpose, various generalizations of the Choquet integral were tested and a set of over a thousand aggregating functions not related to the Choquet integral was verified. In the section on numerical experiments, we indicate the classes of functions and their detailed parameters that work best in terms of the identification of SZ. The dataset applied in this study included data gathered from 40 subjects, i.e., 20 schizophrenic patients and 20 HCs. The Granger causality (GC; Granger, 1969) concept had been applied to particular EEG bands to achieve functional brain connectivity measures. The collected measurements were analyzed using a minimum spanning tree (MST; Stam et al., 2014). The global MST parameters obtained in the analysis were chosen as features in the classified dataset. Applying the MST algorithms enabled grasping the backbone structure of the brain network with only the strongest connections included (González et al., 2016; Van Dellen et al., 2016). Using MST ensured that the link between nodes was not based on an arbitrarily set connectivity strength threshold, which allowed avoiding the bias in network density computations (Tewarie et al., 2015). In general, the MST parameters were chosen because this method lacks some theoretical and mathematical problems incorporated to more typical network organization indicators based on the small-worldness approach and, above all, because the authors wanted to refer to the concept of SZ as a disconnection disease in which pathology of neuronal integration is not isolated only to selected regions or type of synchronizations but has a global dimension. The MST enables the characterization of the global, whole-brain network.

## Materials and Methods

### Participants

Twenty patients, who met the *DSM-5* (*Structured Clinical Interview for DSM-5*) criteria for SZ, were involved from the Department of Psychiatry, Psychotherapy and Early Intervention, Medical University of Lublin. Additionally, the other criteria were as follows: age over 18 years; minimum 10 years of regular education; not more than 5 psychiatric hospitalizations associated with exacerbation of psychosis; no markers of structural brain abnormalities visualized on MRI, indicative of surviving craniocerebral trauma or neurovascular episodes; and lack of serious somatic diseases needing intense pharmacotherapy that would impact the EEG recordings. During testing, all patients were on stable doses of atypical antipsychotics. Using anticholinergic agents, benzodiazepines, and mood stabilizers up to 3 months before the assessment was an exclusionary factor for all participants. The patients participated in the study during the last week of psychiatric hospitalization, after obtaining a significant clinical and functional improvement, being fully able to give consent and undergo EEG examination. The control group consisted of HC, demographically matched to the clinical group, who were chosen from the local community. Additionally, HC had no history of psychiatric diagnoses, per the *Structured Clinical Interview for DSM-5*, brain disease, or neurological injury as well as no family history of psychosis. All patients consented to the study in accordance with the protocol approved by the Bioethical Commission of the Medical University of Lublin. The Commission also validated the methods used in the study. Demographical and clinical data are presented in Table 1. The groups did not differ significantly in terms of age (SZ = 34.41, *SD* = 8.41; HC = 31.63, *SD* = 6.42), number of the years of education (SZ = 12.43, *SD* = 2.94; HC = 14.87, *SD* = 1.68), and gender (SZ = 50% of men; HC = 50% of men). In the SZ group, the duration of illness lasted for about 12 years.

**TABLE 1:** Demographic and clinical data of research groups.

	***SZ* *(n = 20) M (SD)***	***HC* *(n = 20) M (SD)***	***z value or χ^2^***	***p***
Age (years)	32.41 (8.41)	31.63 (6.42)	0.16	0.91
Education (years)	12.43 (2.94)	14.87 (1.68)	−1.12	0.45
Sex (% male)	50	50	0	1
Duration of illness (years)	12.1 (9.43)			
Number of hospitalizations	2.25 (2.65)			
Risperidone equivalents	4.66 (1.76)			

### Data Acquisition

Using a 21-scalp position, electro-cap electroencephalograph (Electro-Cap International Inc., OH, United States) and Ag/AgCl disk electrodes, in 10 min of resting-state, EEG data were recorded for each participant. Electrodes were distributed according to the 10–20 International system (Fp1, Fp2, F3, F4, C3, C4, P3, P4, O1, O2, A1, A2, F7, F8, T3, T4, T5, T6, Fz, Pz, and, Cz). Subjects were seated with eyes closed and restricted head movement. The electrode impedances were kept below 5 k, and the data were filtered from 0.5 to 70 Hz (with active notch filter set at 50 Hz) when the sampling rate was 512 Hz. The data were exported into ASCII format after recording. Post-processing procedures were carried out in the EEGLAB program, which is a MATLAB toolbox. First, the signal was filtered using the bandpass filter at 0.5–45 Hz (second-order Butterworth filter). Second, the reference was changed offline into the averaged. Next, from the processed signal, 25 epochs lasting for 8 s (4,096 samples) without artifacts were extracted for each patient by a clinical neurophysiologist. Last, EEG signals were divided into six frequency bands using finite impulse response filters: the delta (0.5–4 Hz), theta (4–8 Hz), low alpha (8–10 Hz), high alpha (10–12 Hz), beta (13–30 Hz), and gamma (30–45 Hz).

### Data Processing

Several steps were involved in the data processing procedure. Feature extraction was associated with the calculation of functional brain connectivity (FC) measures separately for particular electrodes and each EEG frequency band. The FC was calculated to indicate the statistical dependence between the spatially distributed neurophysiological time series such as EEG signals stemming from separate units of a nervous system (Cheng et al., 2015). There were several metrics used in assessing FC strength, such as classical measures (e.g., Pearson’s correlation coefficient, cross-correlation function, or coherence), phase synchronization indexes (e.g., phase lag index or phase-locking value), and GC measures. We chose the classical linear GC. The idea of GC (Granger, 1969) was based on the assumption that having two simultaneously determined signals (X and Y), the signal X could be better explained using information from the signal Y than using only information from the signal X. In such a situation, signal Y could be specified as “causal” to signal X. The GC measure is widely applied as a statistical tool to detect the influence of particular system components (Nolte et al., 2010). For the GC calculation, we used the Matlab MVGC toolkit (Sackler Centre for Consciousness Science, University of Sussex, Brighton, United Kingdom; Barnett and Seth, 2014), which was based on advanced VAR (vector autoregressive) model theory. To optimize auto-covariance delays, the Akaike information criterion was used to estimate the optimal model order. MVGC algorithms were used to convert EEG signals into auto-covariance data. The observed auto-covariance sequences were then subjected to paired spectral GC. In the case study, the calculated GC measures were used in the next feature extraction procedure step consisting of deriving MST. The MST was built based on Kruskal’s algorithm. First, the weight of all edges was sorted, and then the stronger edges were connected, i.e., those with the highest connectivity values, eliminating those that form loops. These stages were repeated as many times as necessary until the final tree had 19 nodes and 18 edges, which corresponded to the total number of electrodes used in our EEG recording. The MST metrics were generated separately for each frequency band.

Global MST parameters were used as the features for the classification procedure, which included the following:

• maximal degree, a maximal degree in the MST tree,
• maximal BC, maximal betweenness centrality in the MST tree,
• leaf fraction (Lf), the ratio between leaf vertex number (further denoted as L) and the total vertex number,
• diameter (d), the longest distance between any two vertices in the MST tree,
• hierarchy (Th), the measure describing the optimality of the tree topology.

After the feature extraction procedure, the classification was done using classical classifiers.

### A General Processing Scheme

The classification procedure was performed to assign observations into one of two classes: schizophrenic patient and HC. Several classical classification methods were used: cubic SVM, linear SVM, decision tree, logistic regression, multilayer perceptron (MLP), random forest, and k-nearest neighbors (kNN). Data were split into training and testing datasets based on the 80:20 ratio.

The results of this classification process, in the form of the probabilities of belonging to considered classes, were taken as the inputs to the aggregation functions. The next step of the analysis procedure was to generate fuzzy measure density values to apply aggregation operators. The aggregation operators allow combining the predictions of multiple classifiers to further improve the results. The fuzzy measures can be interpreted as the degree of trust (weights or level of importance) to predictions of the individual classifier.

In general, there are several methods of fuzzy measure generation, such as expert assumption, optimization, and the heuristic one. In our study, the cross validation-based heuristic one was applied. N-fold cross-validation was run on the training set to obtain a density measure for a classifier.

**FIGURE 1:**
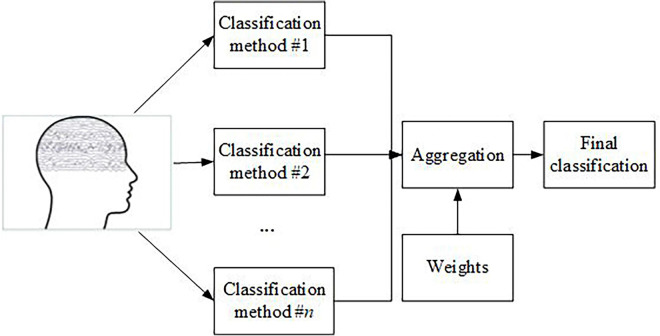
A general classification scheme based on an aggregation process.

It is a well-known fact that the results of different classifications can be aggregated. This situation can be easily illustrated by the example of various sports competitions held in the form of a Grand Prix cycle, where the points are added together to determine the final winner. EEG signal-based features can be similarly aggregated. In general, the results obtained using different kinds of classifiers can be added, averaged, or, generally speaking, transformed with the help of different kinds of aggregation operators. Typical operators are median, minimum, and maximum functions, etc. The general scheme of classification using aggregation methods is presented in Figure 1. It is worth noting that the values that are input to the aggregation operator can change the distances between the training and testing representation of an EEG signal in the case of *k*-nearest neighbor-based classifiers, likelihoods of belonging to a class in the case of neural network-like methods, etc. Here, it is worth stressing that the weights presented in this diagram can be obtained from experts but also based on the quality of classification of individual classifiers (e.g., their accuracy measures).

### Aggregation of Classifiers Using the Choquet Integral and Its Extensions

One of the best known and most efficient classifiers is the Choquet integral. Hence, let us recall the main properties of the fuzzy measure, Choquet integral, and its generalizations. Let us denote a set as *X*. Then *P(X)* = *2*^*X*^ is a family of all its subsets. *2*^*X*^ is a σ-algebra; i.e., the empty set belongs to it, the complement of a set belonging to σ-algebra belongs to it, and the sum of countable many sets from the σ-algebra also belongs to it. Generally speaking, in the context of classification tasks, the elements of the set *X* are the individual classifiers (methods, parts of the images under consideration, etc.). In the context of this specific study, they are particular classifiers, see the experimental section for details of the methods. These classifiers are denoted as *x*_1_,*x*_*n*_,*n*1. Now, one can define (Sugeno, 1974) a fuzzy measure as a set function *g*:*P*(*X*)→*ℝ* satisfying the following conditions:


(1) g⁢(∅)=0⁢and⁢ g⁢(X)=1

(2) g⁢(U)≤g⁢(W),U⊂W,U, W∈P⁢(X)

(3) $$\lim_{n\to \infty }\text{g}\,\left({\text{U}}_{n}\right)=\text{g}\,\left(\lim_{\left(n\to \infty \right)}{\text{U}}_{n}\right)$$

Here, {*U*_*n*_}, *n* = 1, 2, … means increasing set sequence. Recall that Sugeno λ-fuzzy measure realizes the above conditions and


(4) g⁢(U∪W)=g⁢(U)+g⁢(W)+λ⁢g⁢(U)⁢g⁢(W)

with λ >− 1. Here, *U* and *W* are not overlapping. In addition, we have


(5) $$\text{g}\,\left({\text{U}}_{i+1}\right)=\text{g}\,\left({\text{U}}_{i}\right)+{\text{g}}_{i+1}+\lambda \,\text{g}\,\left({\text{U}}_{i}\right)$$

for *U*_*i*_={*x*_1_,…,*x*_*i*_}, *U*_*i*+1_={*x*_1_,…,*x*_*i*+1_}. The following notation is used commonly: *g_*i*_ = g*({*x*_*i*_}), *i* = 1*, …, n*. Now, let us introduce a function *h*(*x*) and let the series *h*(*x*_*i*_), *i* = 1*, …, n*, be ordered non-increasingly and let *h*(*x_*n*__+_*_1_) = 0. In the context of this study, the function *h*(⋅) represents a value of classifier describing the probability of belonging to a specific class. Next, the *U_i* set is, in fact, only an abstract object. The real importance has the value of *g*(*U*_*i*_) appearing in (5) which can be easily found recursively starting from the values of *g*_*i*_. The value *g*_*i*_ represents a significance (or importance) of a particular classifier *x*_*i*_. Its value can be commonly defined twofold: (1) based on the opinions of experts and (2) based on initial tests. In this study, we applied the second method. Finally, *n* is a number of classifiers. The last parameter to be found is λ, which can be obtained from the following equation:


(6) $$1+\lambda =\prod_{i=1}^{n}\left(1+\lambda \,g_{i}\right),g_{i}=g\,\left(\left\{x_{i}\right\}\right)$$

see Sugeno (1974).

For such assumptions, the Choquet integral is defined as


(7) $$\text{C}=\sum_{i=1}^{n}\left(\text{h}\,\left({\text{x}}_{i}\right)-\text{h}\,\left({\text{x}}_{i+1}\right)\,\text{g}\,\left({\text{U}}_{i}\right)\right)$$

From this function, many generalizations and extensions can be delivered as follows:


(8) $${\text{C}}_{M}=\sum_{i=1}^{n}\text{M}\,\left(\text{h}\,\left({\text{x}}_{i}\right)-\text{h}\,\left({\text{x}}_{i+1}\right),\text{g}\,\left({\text{U}}_{i}\right)\right)$$

and for any *t*-norm M (⋅,⋅), see Lucca et al. (2014),


(9) $${\text{C}}_{\mathrm{FM}}=\text{min}\,\left(\sum_{i=1}^{n}\text{M}\,\left(\text{h}\,\left({\text{x}}_{i}\right)-\text{h}\,\left({\text{x}}_{i+1}\right),\text{g}\,\left({\text{U}}_{i}\right)\right),1\right)$$

(Lucca et al., 2014, 2015),


(10) $${\text{C}}_{\mathrm{CM}}=\sum_{i=1}^{n}\left(\text{M}\,\left(\text{h}\,\left({\text{x}}_{i}\right),\text{g}\,\left({\text{M}}_{i}\right)\right)-\text{M}\,\left(\text{h}\,\left({\text{x}}_{i+1}\right),\text{g}\,\left({\text{U}}_{i}\right)\right)\right)$$

see (Lucca et al., 2017), *C*_*Min*_ (Lucca et al., 2015), where the role of the function *M* is played by the minimum, or *C*_*O*_ [see (Lucca et al., 2016)] with a so-called overlap function under the integral sign. Newer functions were proposed in Karczmarek (2018) and Karczmarek et al. (2019a). They are


(11) $$C_{\mathrm{MC}}=\sum_{i=1}^{n}(\text{M}\left(\text{h}\left({\text{x}}_{i}\right),\text{g}\left({\text{U}}_{i}\right)\right)-\text{M}\left(\text{h}\left({\text{x}}_{i+1}\right),\text{g}\left({\text{U}}_{i}\right)\right)+\text{M}\left(\text{h}\left({\text{x}}_{i}\right)-\text{M}\left({\text{x}}_{i+1}\right),\text{g}\left({\text{U}}_{i}\right)\right))$$

(12) $${\text{C}}_{\mathrm{MMin}}=\sum_{i=1}^{n}\text{M}\,\left(\text{min}\,\left(\text{h}\,\left({\text{x}}_{i}\right),\text{g}\,\left({\text{U}}_{i}\right)\right)-\text{min}\,\left(\text{h}\,\left({\text{x}}_{i+1}\right),\text{g}\,\left({\text{U}}_{i}\right)\right),\text{g}\,\left({\text{U}}_{i}\right)\right)$$

(13) $${\text{C}}_{\mathrm{MMin2}}=\sum_{i=1}^{n}\text{M}\,\left(\text{min}\,\left(\text{h}\,\left({\text{x}}_{i}\right),\text{g}\,\left({\text{U}}_{i}\right)\right),\text{min}\,\left(\text{h}\,\left({\text{x}}_{i+1}\right),\text{g}\,\left({\text{U}}_{i}\right)\right)\right)$$

(14) $${\text{C}}_{\mathrm{MinM}}=\sum_{i=1}^{n}\text{min}\,\left(\text{M}\,\left(\left(\text{h}\,\left({\text{x}}_{i}\right),\text{g}\,\left({\text{U}}_{i}\right)\right)\right),\text{M}\,\left(\text{h}\,\left({\text{x}}_{i+1}\right),\text{g}\,\left({\text{U}}_{i}\right)\right)\right)$$

and the integrals inspired by some numerical analysis formulae such as


(15) $${\text{C}}_{\mathrm{D1}}=\sum_{i=1}^{n}\text{M}\,\left(\text{h}\,\left({\text{x}}_{i-1}\right)-\text{h}\,\left({\text{x}}_{i+1}\right),\text{g}\,\left({\text{U}}_{i}\right)\right)$$

(16) $${\text{C}}_{\mathrm{D2}}=\sum_{i=1}^{n}\text{M}\,\left(\text{h}\,\left({\text{x}}_{i-1}\right)+\text{h}\,\left({\text{x}}_{i+1}\right)-\text{h}\,\left({\text{x}}_{i}\right),\text{g}\,\left({\text{U}}_{i}\right)\right)$$

and


(17) $${\text{C}}_{\mathrm{D3}}=\sum_{i=1}^{n}\text{M}\,\left(\frac{\text{h}\,\left({\text{x}}_{i-1}\right)+\text{h}\,\left({\text{x}}_{i+1}\right)}{\text{h}\,\left({\text{x}}_{i}\right)},\text{g}\,\left({\text{U}}_{i}\right)\right)$$

It is worth noting that *M*(⋅,⋅) can be any triangular norm which, as an intersection or conjunction operator in many application areas, is a counterpart to a classic product operator appearing in the original Choquet integral.

## Results

### Individual Classifiers

**FIGURE 2:**
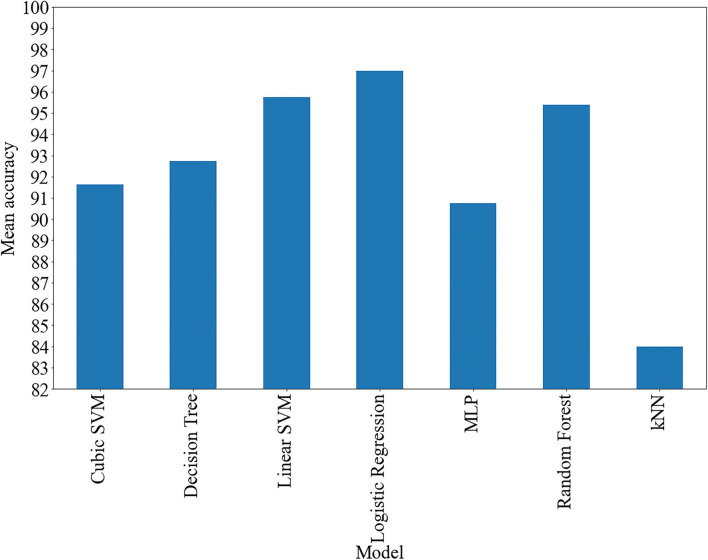
Average of accuracies of separate classifiers.

In this study, we described particular classifiers that were considered in the series of numerical experiments and determined their accuracy. We applied the following classical machine learning models: SVM with linear and cubic kernels, logistic regression, kNN, decision tree, random forest, and MLP. The classical machine learning models were used due to a low number of observations available for training and testing. In order to obtain the fuzzy density that is necessary for aggregation, the following approach can be adapted. According to the holdout validation procedure, the data were split into training and validation subsets, where 20% of the dataset was used for validation. The fuzzy density was calculated as a mean accuracy measure obtained in the process of a fivefold cross-validation run using the training data. The resulting classification quality of separate models was tested on the validation subset after fitting the models on the complete training set. The classification accuracy values obtained with separate classifiers are presented in Figure 2.

### Aggregation of Classifiers

The experimental results of the aggregation scheme used for the classifiers discussed in the previous section, namely decision tree, k-nearest neighbor, quadratic SVM, cubic SVM, linear SVM, logistic regression, random forest, and MLP, are discussed. The accuracies of the individual classifiers obtained in the initial series of experiments are the input to establish the fuzzy measure densities *g*_*i*_. The values of the function *h* are the results of the classification of testing elements being the probabilities of belonging to the two classes, namely, healthy and SZ patients. The validation procedure described in the previous section was repeated 200 times. After each run, a value of fuzzy density and 8 probability vectors (20% out of 40 observations) were obtained per classification model. The details of aggregation algorithm implementation required a single estimation of classification accuracy per model and aggregation method. Hence, all 1,600 (200 × 8) classification results were analyzed. It is worth noting that the models were fitted and the fuzzy densities were obtained independently in every separate run of the experiment. In the series of experiments, we have evaluated 25 classes (families) of popular and commonly considered in the literature triangular norms (Alsina et al., 2006, page 72). The monograph can be treated as a compendium of the *t*-norms to be applied in more advanced aggregation operators. In this particular approach, the *t*-norms serve as the integer functions M with parameters −10, −9.9, …, 0, …, 9.9, 10, but only if the parameter is in the range allowed for the *t*-norm. Such a choice of the parameter range seems to be optimal and emphasizes the most important properties of each of the *t*-norm classes. The maximal accuracy was obtained for the classifier *C*_*D2*_ for triangular norm from the family no. 8, namely


(18) $$\text{M}\,\left(x,y\right)=\frac{\text{max}\,\left(\alpha^{2}\,\text{xy}-\left(1-\text{x}\right)\,\left(1-\text{y}\right),0\right)}{\alpha^{2}-{\left(\alpha -1\right)}^{2}\,\left(1-\text{x}\right)\,\left(1-\text{y}\right)},\alpha >0$$

**FIGURE 3:**
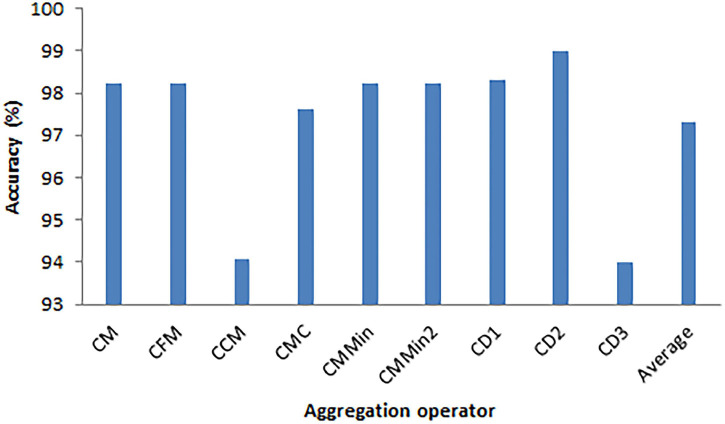
The accuracies were obtained with the function (18) and aggregation operator *C*_*D2*_.

In this case, the best option to choose is α = 0.1. The plot illustrating the recognition rate for the combination of *C*_*D2*_ and the function (18) is given in Figure 3. It is obvious that satisfying accuracy can be obtained only for relatively small values of the parameter α.

It is important to understand the process of calculation of the value of the aggregation function. Let us consider, for example, the process of *C*_*D2*_ finding. Here, the individual classifiers *x*_*i*_,*i* = 1,…,*n* = 5 (five classifiers are discussed in this experiment) should be analyzed, and for their significance measures (simply, weights) *g*_*i*_ = *g*({*x*_*i*_}), see Figure 2. Next, the parameter λ appearing in (6) is calculated. Based on the value of λ, the values of *g*(*U*_*i*_) appearing in Eq. (5) are found recursively. Next, using the values of *h*(*x*_*i*−1_), which are the likelihoods of belongings of a given probe to a specific class, the final sum (16) can be obtained, taking into account that *M*(⋅,⋅) is any *t*-norm, in particular a function given by (19).

Very good results were obtained also for the aggregation functions *C*_*M*_ and very similar *C*_*FM*_. Maximal yielded values were 98.81% for the function number 12 serving as integer function and α = 0.2. The formula of the *t*-norm is as follows:


(19) $$\text{M}\,\left(x,y\right)=\text{max}\,\left(1-{\left({\left(1-\text{x}\right)}^{\alpha }+{\left(1-\text{y}\right)}^{\alpha }\right)}^{\frac{1}{\alpha }},0\right)\,\alpha >0$$

Table 2 supplements the above discussion by showing for which triangular norms and their parameters the classification rate exceeding 98.81% was reached.

**TABLE 2:** Triangular norms and their parameters for the results.

**Aggregation function**	**Number of *t*-norm family**	**α**
*C*_*M*_	12	0.2
*C*_*FM*_	12	0.2
*C*_*D2*_	2	0.5
*C*_*D2*_	8	0.1, 0.2
*C*_*D2*_	11	2.1, 2.4
*C*_*D2*_	13	1.2, 1.3
*C*_*D2*_	14	2.1, 2.4
*C*_*D2*_	25	4.4, 5, 5.1
Average	8	0.1

**FIGURE 4:**
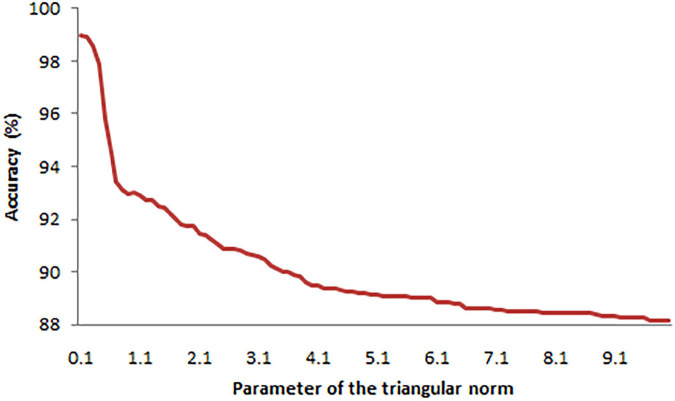
Averages of accuracies achieved with top aggregation functions.

It is worth stressing that the best average result among the operators *C*_*M*_, *C*_*FM*_, *C*_*CM*_, *C*_*MC*_, *C*_*MMin*_, *C*_*MMin2*_, *C*_*D1*_, *C*_*D2*_, and *C*_*D3*_ was also obtained for the triangular norm no. 8 given by the formula (18) and its parameter α = 0.1. The plot presenting the values of the combination of all the aggregation functions with this *t*-norm and parameter is presented in Figure 4. It is obvious that the function (18) works well with almost all of the aggregation operators except *C*_*CM*_ and *C*_*D3*_.

As a supplement to the results, it is worth noting that Table 3 presents the information for which the best results of the *t*-norms were obtained by aggregation operators. It can help match *t*-norms and generalizations of the Choquet integral by experts conducting similar research. For instance, function no. 4, see Alsina et al. (2006), works well when it is combined with a few Choquet integral-based operators.

**TABLE 3:** The best choices of *t*-norms for various generalizations of the Choquet integral.

**Aggregation function**	**Number of *t*-norm family**
*C*_*M*_	12
*C*_*FM*_	12
*C*_*CM*_	11, 14
*C*_*MC*_	11, 14
*C*_*MMin*_	4, 12
*C*_*MMin2*_	4, 12
*C*_*D1*_	4, 13
C_*D2*_	8
C_*D3*_	4

Finally, what should be emphasized, we have conducted the same tests for over 1,000 functions that are not Choquet-like integrals. The functions that were used in this competition were selected based on the studies by Alsina et al. (2006), Beliakov et al. (2007); Grabisch et al. (2009), and Calvo et al. (2012). The best results were obtained for the ordinary weighted averaging operator at the level of 98.81%.


(20) $$\text{OWA}\,\left({\text{x}}_{1},\ldots ,{\text{x}}_{n}\right)=\sum_{j=1}^{n}\omega_{j}\,{\text{y}}_{j}$$

where *y*_*j*_ is the *j*-th largest of the *x*_*i*_, and the weights are *ω_1_* = 1, *ω_2_* = 1 – $\frac{1}{n}$, …, *ω_*n*_* = $\frac{1}{n}$with or without normalization to their sum. Despite it being a very good result, it is obvious that it is hard to find the function giving the results more satisfying than Choquet integral-based operators. Moreover, to find the proper form of OWA, similar to the Choquet integral case, a proper heuristic can be used.

## Conclusion and Future Work

In this study, we have indicated the most appropriate operator aggregating the results of binary classification of patients to efficiently distinguish individuals with SZ and healthy subjects using a set of neural network organization features extracted from EEG-based functional connectivity measures. A series of both types of functions, generalizations of the Choquet integral and other aggregating functions, have been verified to determine the classes of functions and their parameters, which are the most effective in the classification of SZ. As an input to the main analysis, the results of classification were performed with classical methods such as decision tree, k-nearest neighbor, quadratic SVM, cubic SVM, linear SVM, logistic regression, random forest, and MLP were applied. The original results obtained in the study of classical methods classification reached 97% for logistic regression. Although the initially obtained results were high, we decided to verify if there is the possibility to reach even higher results using the fuzzy-based classifier.

The results prove that applying various classification models in combination with aggregation functions enable further improvement of classification results. This approach allows us to take advantage of the additional knowledge cumulated in the parameters of the trained models.

Detailed results show that several aggregation functions enabled to give promising results [presented in the study as Eqs (9–13) and (15, 16)], which increase the classification result by more than 1%. Among numerous functions evaluated and implemented in the thorough comparison, the best accuracy was reached for the aggregating integral *C*_*D2*_ with triangular norm appearing under the integral sign given by the formula (19). Very good results (classification accuracy higher than 98.8%) were reached also for aggregation functions CM and CFM. It is worth noting that the obtained results occurred to be better than the original accuracy reached with classical methods by 1.81%. Although the obtained improvement is not very high (less than 2%), the overall increase in classification accuracy from 97% (for the best classical classifier) to as high as almost 99% (for the properly selected pre-aggregation operators) is relevant. Nevertheless, we have not done double cross-validation analysis, so this limitation can influence classification accuracy, hence the classification rate could be slightly overestimated. Results show the usefulness of this method especially if the role of aggregation function is an extended version of the Choquet integral. In contrast, the application of aggregation functions could give a relatively better improvement in case of weaker initial individual classification results. In future, we have planned to extend the analysis to consider more phases and stages of SZ. Moreover, we are interested in the application of other classes of aggregation operators and the determination of their weights (significance in the process of aggregation) based on the opinions of medical experts. More theoretically, it is still an interesting as well as difficult task to find the optimal parameters of the integral operators only based on their results according to various classification tasks with no relation to the accuracies or expert opinions. Finally, an application of aggregation techniques in other medical pattern recognition or classification problems will be worth analyzing.

## Data Availability Statement

The raw data supporting the conclusions of this article will be made available by the authors, without undue reservation.

## Ethics Statement

The studies involving human participants were reviewed and approved by the Bioethics Committee at the Medical University of Lublin. The patients/participants provided their written informed consent to participate in this study.

## Author Contributions

MP-W and MK: contributed in methodology, software, validation, writing—original draft preparation, and visualization. PKa: contributed in conceptualization methodology, software, writing—original draft preparation, and visualization. MT: contributed in methodology, software, writing—original draft preparation, and visualization. PKr: contributed in methodology, validation, and writing—original draft preparation. KJ: contributed in methodology and writing—original draft preparation. All authors contributed to the article and approved the submitted version.

## Conflict of Interest

The authors declare that the research was conducted in the absence of any commercial or financial relationships that could be construed as a potential conflict of interest.

## Publisher’s Note

All claims expressed in this article are solely those of the authors and do not necessarily represent those of their affiliated organizations, or those of the publisher, the editors and the reviewers. Any product that may be evaluated in this article, or claim that may be made by its manufacturer, is not guaranteed or endorsed by the publisher.

## References

[B1] Alexander-Bloch A. F. Gogtay N. Meunier D. Birn R. Clasen L. Lalonde F. (2010). Disrupted modularity and local connectivity of brain functional networks in childhood-onset schizophrenia. *Front. Syst. Neurosci.* 4:147. 10.3389/fnsys.2010.00147 21031030 PMC2965020

[B2] Alsina C. Schweizer B. Frank M. J. (2006). *Associative Functions: Triangular Norms and Copulas.* Singapore: World Scientific.

[B3] Anderson A. Cohen M. S. (2013). Decreased small-world functional network connectivity and clustering across resting state networks in schizophrenia: an fMRI classification tutorial. *Front. Hum. Neurosci.* 7:520. 10.3389/fnhum.2013.00520 24032010 PMC3759000

[B4] Anderson D. T. Scott G. J. Islam M. A. Murray B. Marcum R. (2018). “Fuzzy choquet integration of deep convolutional neural networks for remote sensing,” in *Computational Intelligence for Pattern Recognition.* (Cham: Springer), 1–28. 10.1007/978-3-319-89629-8_1

[B5] Baczyński M. Bustince H. Mesiar R. (2017). Aggregation functions: theory and applications, part I. *Fuzzy Sets Syst.* 324 1–2.

[B6] Beliakov G. Pradera A. Calvo T. (2007). *Aggregation Functions: A Guide for Practitioners*, Vol. 221. Heidelberg: Springer.

[B7] Barnett L. Seth A. K. (2014). The MVGC multivariate Granger causality toolbox: a new approach to Granger-causal inference. *J. Neurosci. Methods* 223 50–68. 24200508 10.1016/j.jneumeth.2013.10.018

[B8] Bustince H. Sanz J. A. Lucca G. Dimuro G. P. Bedregal B. Mesiar R. (2016). “Pre-aggregation functions: definition, properties and construction methods,” in *2016 IEEE International Conference on Fuzzy Systems (FUZZ-IEEE).* (Piscataway, NJ: IEEE), 294–300.

[B9] Calvo T. Mayor G. Mesiar R. (2012). *Aggregation Operators: New Trends and Applications*, Vol. 97. Heidelberg: Physica.

[B10] Cheng W. Palaniyappan L. Li M. Kendrick K. M. Zhang J. Luo Q. (2015). Voxel-based, brain-wide association study of aberrant functional connectivity in schizophrenia implicates thalamocortical circuitry. *NPJ Schizophr.* 1 1–8. 10.1038/npjschz.2015.16 PMC4849447 27336032

[B11] Dias C. A. Bueno J. C. Borges E. N. Botelho S. S. Dimuro G. P. Lucca G. (2018). “Using the Choquet integral in the pooling layer in deep learning networks,” in *North American Fuzzy Information Processing Society Annual Conference.* (Cham: Springer), 144–154. 10.1007/978-3-319-95312-0_13

[B12] Dimuro G. P. Fernández J. Bedregal B. Mesiar R. Sanz J. A. Lucca G. (2020). The state-of-art of the generalizations of the Choquet integral: from aggregation and pre-aggregation to ordered directionally monotone functions. *Inform. Fusion* 57 27–43. 10.1016/j.inffus.2019.10.005

[B13] Dimuro G. P. Lucca G. Sanz J. A. Bustince H. Bedregal B. (2017). “CMin-Integral: a Choquet-like aggregation function based on the minimum t-norm for applications to fuzzy rule-based classification systems,” in *International Summer School on Aggregation Operators.* (Cham: Springer), 83–95. 10.1007/978-3-319-59306-7_9

[B14] Dolecki M. Karczmarek P. Kiersztyn A. Pedrycz W. (2016). “Utility functions as aggregation functions in face recognition,” in *2016 IEEE Symposium Series on Computational Intelligence (SSCI).* (Piscataway, NJ: IEEE), 1–6.

[B15] Friston K. J. Frith C. D. (1995). Schizophrenia: a disconnection syndrome. *Clin. Neurosci.* 3 89–97. 7583624

[B16] Gągolewski M. (2015). *Data Fusion: Theory, Methods, and Applications.* Warsaw: Institute of Computer Science, Polish Academy of Sciences.

[B17] Gallos I. K. Galaris E. Siettos C. I. (2021a). Construction of embedded fMRI resting-state functional connectivity networks using manifold learning. *Cogn. Neurodyn.* 15 585–608. 10.1007/s11571-020-09645-y 34367362 PMC8286923

[B18] Gallos I. K. Gkiatis K. Matsopoulos G. K. Siettos C. (2021b). ISOMAP and machine learning algorithms for the construction of embedded functional connectivity networks of anatomically separated brain regions from resting state fMRI data of patients with Schizophrenia. *AIMS Neurosci.* 8 295–321. 10.3934/Neuroscience.2021016 33709030 PMC7940114

[B19] González G. F. Van der Molen M. J. W. Žarić G. Bonte M. Tijms J. Blomert L. (2016). Graph analysis of EEG resting state functional networks in dyslexic readers. *Clin. Neurophysiol.* 127 3165–3175. 10.1016/j.clinph.2016.06.023 27476025

[B20] Grabisch M. Marichal J. L. Mesiar R. Pap E. (2009). *Aggregation Functions (No. 127).* Cambridge: Cambridge University Press.

[B21] Granger C. W. (1969). Investigating causal relations by econometric models and cross-spectral methods. *Econometrica* 37 424–438. 10.2307/1912791

[B22] Green M. F. Horan W. P. Lee J. (2019). Nonsocial and social cognition in schizophrenia: current evidence and future directions. *World Psychiatry* 18 146–161. 10.1002/wps.20624 31059632 PMC6502429

[B23] Guo X. Dominick K. C. Minai A. A. Li H. Erickson C. A. Lu L. J. (2017). Diagnosing autism spectrum disorder from brain resting-state functional connectivity patterns using a deep neural network with a novel feature selection method. *Front. Neurosci.* 11:460. 10.3389/fnins.2017.00460 28871217 PMC5566619

[B24] Heilbronner U. Samara M. Leucht S. Falkai P. Schulze T. G. (2016). The longitudinal course of schizophrenia across the lifespan: clinical, cognitive, and neurobiological aspects. *Harvard Rev. Psychiatry* 24 118–128. 10.1097/HRP.0000000000000092 26954596 PMC5079232

[B25] Huang J. Zhu Q. Hao X. Shi X. Gao S. Xu X. (2018). Identifying resting-state multifrequency biomarkers via tree-guided group sparse learning for schizophrenia classification. *IEEE J. Biomed. Health Inform.* 23 342–350. 10.1109/JBHI.2018.2796588 29994431

[B26] Jonak K. Krukow P. Jonak K. E. Grochowski C. Karakuła-Juchnowicz H. (2019). Quantitative and qualitative comparison of EEG-based neural network organization in two schizophrenia groups differing in the duration of illness and disease burden: graph analysis with application of the minimum spanning tree. *Clin. EEG Neurosci.* 50 231–241. 10.1177/1550059418807372 30322279

[B27] Karczmarek P. (2018). *Selected Problems of Face Recognition and Decision-Making Theory.* Lublin: Wydawnictwo Politechniki Lubelskiej.

[B28] Karczmarek P. Kiersztyn A. Pedrycz W. (2017a). “An evaluation of fuzzy measure for face recognition,” in *International Conference on Artificial Intelligence and Soft Computing.* (Cham: Springer), 668–676. 10.1109/TSMCB.2012.2185693

[B29] Karczmarek P. Kiersztyn A. Pedrycz W. (2017b). On developing Sugeno fuzzy measure densities in problems of face recognition. *Int. J. Mach. Intell. Sens. Signal Process.* 2 80–96. 10.1504/ijmissp.2017.088185

[B30] Karczmarek P. Kiersztyn A. Pedrycz W. (2018). Generalized choquet integral for face recognition. *Int. J. Fuzzy Syst.* 20 1047–1055. 10.1007/s40815-017-0355-5

[B31] Karczmarek P. Kiersztyn A. Pedrycz W. (2019b). “Generalizations of aggregation functions for face recognition,” in *International Conference on Artificial Intelligence and Soft Computing.* (Cham: Springer), 182–192. 10.1007/978-3-030-20915-5_17

[B32] Karczmarek P. Pedrycz W. Kiersztyn A. Dolecki M. (2019a). A comprehensive experimental comparison of the aggregation techniques for face recognition. *Ira. J. Fuzzy Syst.* 16 1–19.

[B33] Karczmarek P. Pedrycz W. Reformat M. Akhoundi E. (2014). A study in facial regions saliency: a fuzzy measure approach. *Soft Comput.* 18 379–391. 10.1007/s00500-013-1064-0

[B34] Keefe R. S. (2019). Why are there no approved treatments for cognitive impairment in schizophrenia? *World Psychiatry* 18 167–168. 31059617 10.1002/wps.20648 PMC6502426

[B35] Klauser P. Baker S. T. Cropley V. L. Bousman C. Fornito A. Cocchi L. (2017). White matter disruptions in schizophrenia are spatially widespread and topologically converge on brain network hubs. *Schizophr. Bull.* 43 425–435. 10.1093/schbul/sbw100 27535082 PMC5605265

[B36] Klement E. P. Mesiar R. Pap E. (2000). *Triangular Norms.* Dordrecht: Springer.

[B37] Krukow P. Jonak K. Grochowski C. Plechawska-Wójcik M. Karakuła-Juchnowicz H. (2020). Resting-state hyperconnectivity within the default mode network impedes the ability to initiate cognitive performance in first-episode schizophrenia patients. *Prog. Neuropsychopharmacol. Biol. Psychiatry* 102:109959. 10.1016/j.pnpbp.2020.109959 32376341

[B38] Krukow P. Jonak K. Karakuła-Juchnowicz H. Podkowiński A. Jonak K. Borys M. (2018). Disturbed functional connectivity within the left prefrontal cortex and sensorimotor areas predicts impaired cognitive speed in patients with first-episode schizophrenia. *Psychiatry Res.* 275 28–35. 10.1016/j.pscychresns.2018.03.001 29526598

[B39] Krukow P. Karakuła-Juchnowicz H. Juchnowicz D. Morylowska-Topolska J. Flis M. Jonak K. (2017). Processing speed is associated with differences in IQ and cognitive profiles between patients with schizophrenia and their healthy siblings. *Nordic J. Psychiatry* 71 33–41. 10.1080/08039488.2016.1204469 27387772

[B40] Kwak K. C. Pedrycz W. (2004). Face recognition using fuzzy integral and wavelet decomposition method. *IEEE Trans. Syst. Man Cybernet. Part B* 34 1666–1675. 10.1109/tsmcb.2004.827609 15462434

[B41] Kwak K. C. Pedrycz W. (2005). Face recognition: a study in information fusion using fuzzy integral. *Pattern Recognit. Lett.* 26 719–733.

[B42] Liu H. Zhang T. Ye Y. Pan C. Yang G. Wang J. (2017). A data driven approach for resting-state EEG signal classification of schizophrenia with control participants using random matrix theory. *arXiv* [preprint], Avaliable online at: https://arxiv.org/abs/1712.05289 (accessed June 30, 2021).

[B43] Lucca G. Sanz J. A. Dimuro G. P. Bedregal B. Asiain M. J. Elkano M. (2017). CC-integrals: Choquet-like copula-based aggregation functions and its application in fuzzy rule-based classification systems. *Knowl. Based Syst.* 119 32–43. 10.1016/j.knosys.2016.12.004

[B44] Lucca G. Sanz J. A. Dimuro G. P. Bedregal B. Bustince H. (2016). “Pre-aggregation functions constructed by CO-integrals applied in classification problems,” in *Proceedings of IV CBSF.* (New York, NY: AMC), 1–11.

[B45] Lucca G. Sanz J. A. Dimuro G. P. Bedregal B. Mesiar R. Kolesárová A. (2015). “The notion of pre-aggregation function,” in *International Conference on Modeling Decisions for Artificial Intelligence.* (Cham: Springer), 33–41. 10.1007/978-3-319-23240-9_3

[B46] Lucca G. Vargas R. Dimuro G. P. Sanz J. Bustince H. Bedregal B. (2014). “Analysing some t-norm-based generalizations of the Choquet integral for different fuzzy measures with an application to fuzzy rule-based classification systems,” in *ENIAC 2014, Encontro Nac. Intelig. Artificial e Computacional.* (São Carlos: SBC), 508–513.

[B47] Nolte G. Ziehe A. Krämer N. Popescu F. Müller K. R. (2010). “Comparison of Granger causality and phase slope index,” in *Proceedings of Workshop on Causality: Objectives and Assessment at NIPS 2008*, Vol. 6, 267–276.

[B48] Pantelis C. Yücel M. Bora E. Fornito A. Testa R. Brewer W. J. (2009). Neurobiological markers of illness onset in psychosis and schizophrenia: the search for a moving target. *Neuropsychol. Rev.* 19 385–398. 10.1007/s11065-009-9114-1 19728098

[B49] Parvinnia E. Sabeti M. Jahromi M. Z. Boostani R. (2014). Classification of EEG Signals using adaptive weighted distance nearest neighbor algorithm. *J. King Saud Univers. Comput. Inform. Sci.* 26 1–6. 10.1016/j.jksuci.2013.01.001

[B50] Phang C. R. Noman F. Hussain H. Ting C. M. Ombao H. (2019). A multi-domain connectome convolutional neural network for identifying schizophrenia from EEG connectivity patterns. *IEEE J. Biomed. Health Informat.* 24 1333–1343. 10.1109/JBHI.2019.2941222 31536026

[B51] Plis S. M. Hjelm D. R. Salakhutdinov R. Allen E. A. Bockholt H. J. Long J. D. (2014). Deep learning for neuroimaging: a validation study. *Front. Neurosci.* 8:229. 10.3389/fnins.2014.00229 25191215 PMC4138493

[B52] Rosen W. G. Mohs R. C. Johns C. A. Small N. S. Kendler K. S. Horvath T. B. (1984). Positive and negative symptoms in schizophrenia. *Psychiatry Res.* 13 277–284. 6596585 10.1016/0165-1781(84)90075-1

[B53] Rutkowska D. Kurach D. Rakus-Andersson E. (2020). “Face recognition with explanation by fuzzy rules and linguistic description,” in *International Conference on Artificial Intelligence and Soft Computing.* (Cham: Springer), 338–350.

[B54] Sabeti M. Katebi S. D. Boostani R. Price G. W. (2011). A new approach for EEG signal classification of schizophrenic and control participants. *Expert Syst. Appl.* 38 2063–2071. 10.1016/j.eswa.2010.07.145

[B55] Sabeti M. Sadreddini M. Price G. (2007). “Fuzzy accuracy-based classifier systems for EEG classification of schizophrenic patients,” in *In First Joint Congress on Fuzzy and Intelligent Systems Ferdowsi.* (Iran: University of Mashhad), 29–31.

[B56] Sheffield J. M. Repovs G. Harms M. P. Carter C. S. Gold J. M. MacDonald A. W. III (2015). Fronto-parietal and cingulo-opercular network integrity and cognition in health and schizophrenia. *Neuropsychologia* 73 82–93. 10.1016/j.neuropsychologia.2015.05.006 25979608 PMC4505838

[B57] Shen H. Wang L. Liu Y. Hu D. (2010). Discriminative analysis of resting-state functional connectivity patterns of schizophrenia using low dimensional embedding of fMRI. *Neuroimage* 49 3110–3121. 10.1016/j.neuroimage.2009.11.011 19931396

[B58] Shim M. Hwang H. J. Kim D. W. Lee S. H. Im C. H. (2016). Machine-learning-based diagnosis of schizophrenia using combined sensor-level and source-level EEG features. *Schizophr. Res.* 176 314–319. 10.1016/j.schres.2016.05.007 27427557

[B59] Shim M. Kim D. W. Lee S. H. Im C. H. (2014). Disruptions in small-world cortical functional connectivity network during an auditory oddball paradigm task in patients with schizophrenia. *Schizophr. Res.* 156 197–203. 10.1016/j.schres.2014.04.012 24819192

[B60] Silvana M. Akbar R. Audina M. (2018). “Development of classification features of mental disorder characteristics using the fuzzy logic Mamdani method,” in *2018 International Conference on Information Technology Systems and Innovation (ICITSI).* (Bandung: IEEE), 410–414.

[B61] Skudlarski P. Jagannathan K. Anderson K. Stevens M. C. Calhoun V. D. Skudlarska B. A. (2010). Brain connectivity is not only lower but different in schizophrenia: a combined anatomical and functional approach. *Biol. Psychiatry* 68 61–69. 10.1016/j.biopsych.2010.03.035 20497901 PMC2900394

[B62] Sporns O. Tononi G. Kötter R. (2005). The human connectome: a structural description of the human brain. *PLoS Comput. Biol.* 1:e42. 10.1371/journal.pcbi.0010042 16201007 PMC1239902

[B63] Stam C. J. Tewarie P. Van Dellen E. van Straaten E. C. Hillebrand A. Van Mieghem P. (2014). The trees and the forest: characterization of complex brain networks with minimum spanning trees. *Int. J. Psychophysiol.* 92 129–138. 10.1016/j.ijpsycho.2014.04.001 24726900

[B64] Sugeno M. (1974). *Theory of fuzzy Integral and Its Applications.* Dissertation, Tokyo: Tokyo Institute of Technology.

[B65] Szöke A. Trandafir A. Dupont M. E. Meary A. Schürhoff F. Leboyer M. (2008). Longitudinal studies of cognition in schizophrenia: meta-analysis. *Br. J. Psychiatry* 192 248–257. 10.1192/bjp.bp.106.029009 18378982

[B66] Tewarie P. van Dellen E. Hillebrand A. Stam C. J. (2015). The minimum spanning tree: an unbiased method for brain network analysis. *Neuroimage* 104 177–188. 10.1016/j.neuroimage.2014.10.015 25451472

[B67] Uhlhaas P. J. (2013). Dysconnectivity, large-scale networks and neuronal dynamics in schizophrenia. *Curr. Opin. Neurobiol.* 23 283–290. 10.1016/j.conb.2012.11.004 23228430

[B68] Van Dellen E. Bohlken M. M. Draaisma L. Tewarie P. K. van Lutterveld R. Mandl R. (2016). Structural brain network disturbances in the psychosis spectrum. *Schizophr. Bull.* 42 782–789. 10.1093/schbul/sbv178 26644605 PMC4838099

[B69] Van Den Heuvel M. P. Fornito A. (2014). Brain networks in schizophrenia. *Neuropsychol. Rev.* 24 32–48. 24500505 10.1007/s11065-014-9248-7

[B70] van den Heuvel M. P. Sporns O. (2013). Network hubs in the human brain. *Trends Cogn. Sci.* 17 683–696. 10.1016/j.tics.2013.09.012 24231140

[B71] Yager R. R. Kacprzyk J. (eds) (2012). *The Ordered Weighted Averaging Operators: Theory and Applications.* Berlin: Springer Science & Business Media.

[B72] Yan H. Tian L. Wang Q. Zhao Q. Yue W. Yan J. (2015). Compromised small-world efficiency of structural brain networks in schizophrenic patients and their unaffected parents. *Neurosci. Bull.* 31 275–287. 10.1007/s12264-014-1518-0 25813916 PMC5563688

[B73] Zalesky A. Fornito A. Bullmore E. T. (2010). Network-based statistic: identifying differences in brain networks. *Neuroimage* 53 1197–1207. 10.1016/j.neuroimage.2010.06.041 20600983

[B74] Zalesky A. Fornito A. Seal M. L. Cocchi L. Westin C. F. Bullmore E. T. (2011). Disrupted axonal fiber connectivity in schizophrenia. *Biol. Psychiatry* 69 80–89. 21035793 10.1016/j.biopsych.2010.08.022 PMC4881385

[B75] Zhu Q. Huang J. Xu X. (2018). Non-negative discriminative brain functional connectivity for identifying schizophrenia on resting-state fMRI. *Biomed. Eng. Online* 17 1–15. 10.1186/s12938-018-0464-x 29534759 PMC5851331

